# The reported thoracic injuries in Homer's Iliad

**DOI:** 10.1186/1749-8090-5-114

**Published:** 2010-11-19

**Authors:** Efstratios Apostolakis, Georgia Apostolaki, Mary Apostolaki, Maria Chorti

**Affiliations:** 1 Department of Cardiothoracic Surgery. Patras University School of Medicine, Patras, Greece; 2 English Literature. University of Athens. Athens, Greece; 3 Pedagogic Literature. University of Athens. Athens, Greece; 4 Department of Pathology. ''Sismanogleio'' General Hospital of Athens. Athens, Greece

## Abstract

Homer's Iliad is considered to be a prominent and representative work of the tradition of the ancient Greek epic poetry. In this poem Homer presents the battles which took place during the last year of the 10-year lasting Trojan War between Achaeans and Trojans. We wanted to examine the chest wounds, especially those which are described in detail, according to their localization, severity and mortality. Finally, there are reported 54 consecutive thoracic injuries in the Iliad. The mostly used weapons were the spear (63%), the stones (7.4%), the arrow (5.5%) and the sword (5.5%). We divided the injuries according to their severity in mild (those which did not cause serious injury to the victim), medium (those which cause the victim to abandon the battlefield), and severe (those which cause death of the victim). According to this classification, the reported injuries were mild in 11.11%, medium in 18.52%, and severe in the last 70.37% of the reported cases. In other words, 89% of the injuries belong to the medium or severe category of thoracic injury. As far as the mortality of the injuries is concerned, 38 out of 54 thoracic injuries include death, which makes the mortality percentage reach 70.37%. Concerning the "allocation of the roles", the Achaean were in 68% perpetrators and the Trojans in only 32%. In terms of gravity, out of 38 mortal injuries 30 involve a Trojan (78.95%) and the remaining 8 an Achaean (21.05%). The excellent and detailed description of the injuries by Homer, as well as of the symptoms, may reveal a man with knowledge of anatomy and medicine who cared for the injured warriors in the battlefield.

## Introduction

*"...while fighting Idomeneus stabbed at the middle of his chest with the spear, and broke the bronze armour about him which in time before had guarded his body from destruction. He cried out then, a great cry, broken, the spear in him, and fell, thunderously, and the spear in his heart was struck fast but the heart was panting still and **beating to shake the butt end of the spear**. Then and there Ares the huge took his life away from him..." (Book 13, verses 438-444) *[1]

**Figure 1:**
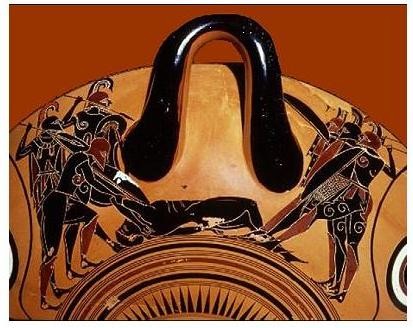
**Amphora representing a lethal battle between the Achaeans and the Trojans over Patroclus' dead body**. The Trojans, presented on the left side, have already despoiled Achilles' famous attire, which Patroclus was wearing, but eventually the Achaeans, presented on the right, claim the corpse. (black-figure drinking cup dated 500 BC, Archaeological Museum of Munich).

The "Iliad" and "Odyssey" of Homer are the foundation stones of classical Greek literature, and therefore also of the literature of Western civilization. Homer was read, memorized and quoted throughout the great age of ancient Greece, and was regarded as the poet who surpassed all others [2]. The Iliad and the Odyssey comprise two of the most important works of classical Greek literature and they have influenced, to a great extent, Western literature. The Iliad, in particular, is considered to be a prominent and representative work of the tradition of the ancient Greek epic poetry. By means of a vivid, unsurpassed description of the war of Troy the poet presents the battles which took place during the last year of this 10-year war (figure 1). In an ambient of insufferable impatience-or even despair-as well as nostalgia for their country, the Trojans faced the Achaeans, the former being exhausted due to the long-lasting siege of the latter. Homer offers the description of a merciless and rabid combat that leads to the destructive, on the part of the Trojans, ending. The poem unravels the story of a war which proves to be a vacillating and inexpedient conflict.

According to Mumford D [2], anger, wrath, aggression, fear and panic constitute the psychological state which characterizes the heroes of Iliad. In this tragically drawn picture, people and Gods are brought into conflict, obeying, however, the rules of an earthly "war game", using namely human weapons of the era, so that both humans and gods would be equal opponents following the same rules of the art of war. Around the bloodshed walls of Troy lethal combats took place, involving hand-to-hand conflicts (b. 2, v. 265-270), (b. 4, v. 134-140), (b. 4, v. 473-488), (b. 5, v. 38-42), (b. 5, v. 79-83), (b. 7, v. 318-322), (b. 8, v. 219-225), (b. 8, v. 268-272), (b. 9, v. 320-329), (b. 11, v. 76-79), (b. 12, v. 15-46), (b. 14, v. 264-265), (b. 21, v. 116-120) [1]. The arms used in these battles were "low-energy" ones, as they are commonly known: arrows, lances, javelins, stones, and bludgeons [3-6]. This meant that the wounds were, in general, non-lethal and the injured usually survived their wounds or, at least, lived for a long time after the injury. Consequently, the wound was "accessible" to their comrade-in-arms and thus the latter could observe and offer a detailed description of it (b. 5, v. 95-100), (b. 5, v. 79-83), (b. 8, v. 257-260), (b. 8, v. 300-308), (b. 11, v. 446-449), (b. 13, v. 437-444), (b. 13, v. 595-600), (b. 15, v. 541-543) [1]. It must have been similarly easy for a skilful writer, such as Homer, to produce extensive descriptions of these wounds. Indeed, the Iliad abounds with such descriptions of wounds of all kinds, ranging from light to instantaneously fatal ones. The latter involve mainly injuries to the head and the torso, and more particularly the chest [3]. This study will focus on the descriptions which especially involve chest injuries caused during the Trojan War. It goes without saying that in such a war there would be thousands of wounds. What would be of interest here is to examine the chest wounds, especially those which are described in detail, be it the wound of a prominent war hero ("Afterwards with Erymas, Amphoteros, and Epaltes, Tlepolemos Damastor's son, Echios and Pyris, Ipheus and Euippos, and Argeas' son Polymelos, all these he felled to the bountiful earth in rapid succession") (b. 16, v. 415-418) [1] or that of an inconspicuous victim.

## Materials and methods

In order to discern the diverse injuries mentioned in the Iliad, a meticulous reading of the whole poem is necessary although in some rhapsodies (books in English translation) -1, 3, 9, 18, 19, and 24- there is no reference to injuries. These rhapsodies include the events which occurred during the "intermissions" of the war. Other rhapsodies, for instance 5, 13, 16 or 12 are characterized as "the most lethal ones" (see additional file 1). For most of the reported injuries there is a reference not only to the method used by the perpetrator to injure his/her victim or the area where the injury occurred but also to other factors, such as the place of origin of the victim and the perpetrator, the nature of the weapon which caused the injury and the outcome of the conflict (b. 2, v. 265-270), (b. 4, v. 134-140), (b. 4, v. 527-531), (b. 5, v. 17-24), (b. 5, v. 38-42), (b. 5, 55-58), (5, 95-100), (b. 8, v. 300-308), (b. 8, v. 320-v. 329), (b. 11, v. 434-438), (b. 11, v. 446-449), (b. 21, v. 116-120) [1]. Limiting the survey to the sole description of the injuries viewed merely as medical cases would definitely undermine the work of this skilful poet. Therefore, in the last column of the table above whole passages from the original text are quoted so that the reader can relish the vivid descriptions of unique beauty as presented by the poet himself.

Concerning the estimation of the gravity and mortality of the thoracic injuries, **t**here is great difficulty either because there is a lack of medical details or because of the lack of continuity in the description of the injury. Homer seldom includes a reference to the therapy following the injury, as in cases 11 and 15. Only in cases of lethal wounds can we infer that the injury was grave. In this survey the injuries are arbitrarily categorized according to a three-level scale: "**mild injuries" **or "(**+)" **are those which did not cause serious injury to the warrior and so he could return to the battlefield. "**Severe injuries" or "(+++)" **are those which cause the victim to fall on the ground. In all these injuries the victim dies instantly. Finally, "**medium injuries" **or "(**++)" **are those which cause the victim to abandon the battlefield without causing death.

## Results

### Injuries according to rhapsodies

From a total of 151 injuries, 54 are injuries of the chest (35, 76%) (See additional file 1). Santos G [3] includes a much smaller percentage in his survey (20%) since the survey mentions 26 chest injuries out of 130. The 54 injuries mentioned in our survey include 53 warriors and two of them involved the same warrior, Diomedes (cases 11 and 13 in additional file 1). Most of the injuries can be found in rhapsody 5 (11 injuries) and then follow rhapsody 16 (7 injuries), rhapsody 11 (6 injuries), rhapsodies 7 and 15 (5 injuries), rhapsodies 4 and 13 (4 injuries), rhapsody 21 (3 injuries), rhapsodies 7, 14, 17 and 20 (2 injuries), and, rhapsody 2 (1 injury).

### Victimizers or perpetrators and victims

**Figure 2:**
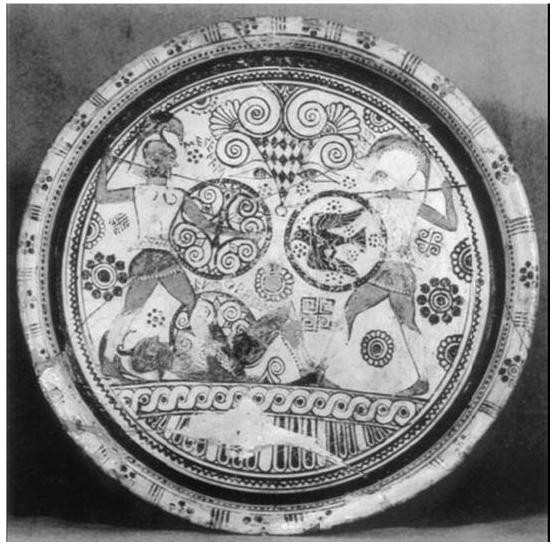
**Amphora dated 610 BC, Archaeological Museum of London**. Menelaus and Hector's combat over Euphorbus' body. The latter, the son of the priest of Apollo Panthos, had wounded Patroclus in his back and then Hector stroke Euphorbus in the chest.

The directness of Homer's language transforms Gods into creatures of this earth and renders the heroes brave or even arrogant. By means of the skilful use of language Homer manages to place the two opponents, the Gods and the mortals, on an equal level in the field of battle: both groups share the same passions. They feel hatred, love and what they desire is to avenge themselves or to win the battle and, consequently, they confront each other with incredible vehemence. From a total of 54 thoracic injuries, 50 include those between two mortals whereas the rest of them are the result of a conflict between two Gods or a God and a mortal. Indeed, in two cases (cases 14 and 15) one semi-God, son of Zeus, Amphitrionades, attacks Hera and Hades. In addition, in cases 53 and 54, Goddess Athena attacks the God of war, Ares, as well as the Goddess of Beauty, Aphrodite. The Gods' partiality for one hero or another was often demonstrated by means of their intervention in the field of battle. A singular example is case 47 (b. 16, v. 791-809) [1], where Apollo strips brave Patroclus of his armour during the battle and, thus, Euphorbus the Trojan manages to strike the latter in the interscapular area. Consequently, Hector exploits Patroclus' vulnerability and strikes him in the inguinal area which causes Patroclus to die (b.16, v. 818-822) [1]. (Figure 2).

### Perpetrator-victim or Homer's "partiality"

Homer's partiality is made apparent in the poem although the reader may expect an impartial presentation of the events. The poet constantly tends to praise the Achaeans' superiority over the Trojans. How else can the analogy between the perpetrators and the victims be explained? One could argue that the Achaean perpetrators excelled in number the Trojan perpetrators only during the last year of the war and this is the reason why Homer's description is "partial". In the epic, the perpetrator was an Achaean in 34 cases and a Trojan in 16 cases. In 4 cases the perpetrator was a God or a semi-God. Concerning the victims, 35 of them were Trojans, 15 were Achaeans and 4 were Gods or semi-Gods.

### Weapons used

There is a wide variety of weapon mentioned in the conflicts, ranging from spears to stones or even the scepter of Odysseus, king of Ithaca. The use of the spear is mentioned in 34 cases of the thoracic injuries (62.96%). The use of the arrow is the second most important weapon which is mentioned in three cases (5.55%), the stone is mentioned in 4 cases (7.40%), the sword in three cases (5.55%), the javelin as well as the sword in two cases (3.70%) and, finally, the hand and the scepter in one case (1.85%) (b. 2, v. 265-270) [1].

### Localization of the thoracic injuries

Detecting and analyzing the injuries is a difficult task since their description is not always precisely reported. Most of the injuries to the back are referred to as "metaphrenon" without mentioning whether they occurred in the interscapular area or at the basis of the thorax (see table). Moreover, in some of the injuries of the upper thorax it is difficult to distinguish between those of the thorax and those of the neck. Some of the injuries, for instance those of the shoulder or the arm, may be categorized as injuries of the thorax since the result of the attack was instant death. The same categorization may also be applied to some of the injuries of the hip or the pelvis. Another difficulty is that some injuries combine two different areas of the body: 3 of them include the thorax and the abdomen (cases 5, 18, 32), two of them appear in the thorax and the shoulder (cases 33 and 38), two injuries include the thorax and the neck (cases 23 and 34) and one includes the thorax and the head (case 26) (see additional file 1). Unfortunately, out of the 46 injuries which relate to the thorax there is a lack of information for 9 injuries (cases 2, 3, 13, 21, 25, 27, 30, 36 and 54 of the additional file 1) and, consequently, their categorization in one of the subcategories in table is rendered difficult. The 37 injuries which remain can be categorized in relation to the area of the body in which they appear in the table.

### The gravity and mortality of the thoracic injuries

According to the aforementioned evaluation of injuries (see "Material and Methods"), the 54 thoracic injuries mentioned in Homer's work can be categorized as follows:

**a. Mild **or **(+)**: 6 cases (11.11%) (The cases 1, 6, 18, 34, 53, 54 of the additional file 1)

**b. Medium **or **(++)**: 10 cases (18.52%) (The cases 2, 11, 13, 14, 15, 23, 27, 29, 47, 49 of the additional file 1)

**c. Severe **or **(+++)**: 38 cases (70.37%) (the cases 3, 4, 5, 7, 8, 9, 10, 12, 16, 17, 19, 20, 21, 22, 24, 25, 26, 28,30, 31, 32, 33, 35, 36, 37, 38, 39, 40, 41, 42, 43, 44, 45, 46, 48, 50, 51, 52 of the additional file 1).

As it is shown in the categories above (see "Material and Methods"), 89% of the injuries belong to the medium or severe category of thoracic injury. As far as the mortality of the injuries is concerned, 38 out of 54 thoracic injuries include death, which makes the mortality percentage reach 70.37%. It should be noted that all serious injuries which result in death are cases of "instant mortality". If we were to divide the mortality of the injuries according to the tribe, the conclusion would be that the Trojans had far more casualties than the Achaeans. Of course, out of the 54 injuries we would have to omit 4 (cases 14, 15, 53, 54) since they involve Gods. Out of the remaining 50, only 16 or 32% of injuries involve a Trojan perpetrator, while the majority (34 cases or 68%) involves an Achaean one. In terms of gravity, out of 38 mortal injuries 30 involve a Trojan (78.95%) and the remaining 8 and Achaean (21.05%). On the contrary, in 12 non-mortal injuries ("light" or "medium") a Trojan perpetrator appears in 8 cases (66.66%) while an Achaean attacker is mentioned in only four (33.33%). Therefore, it can be inferred that although Homer attempts to present the two sides as equally powerful, he rather gives a biased report of the incidents of the War. There are many reasons attributed; firstly, the majority of the Olympian Gods were supporting the Achaeans and they used all means possible to demonstrate their preference. Athena (b. 5, v. 836-837), (b. 8, v. 358-363), (b. 10, v. 482-487), (b. 11, v. 10-12), (b. 15, v. 68-70 and 211-217), Hera (b. 5, v. 784-791), (b. 8, v. 352-356), (b. 15, v. 211-217), Poseidon (b. 15, v. 211-217), Hermes (b. 15, v. 211-217) and Hephaestus (b. 15, v. 211-217) side with the Achaeans (b. 20, v. 33-37) [1]. On the contrary, Zeus (b. 8, v. 352-356), (b. 11, v. 78-79), (b. 15, v. 14-17, v. 68-70, v. 228-235, and v. 254-255), Apollo (b. 7, v. 272), (b. 15, v. 228-235 and v. 254-255), Ares (b. 5, v. 845-860), Aphrodite (b. 5, v. 376-378), Leto and Artemis support the Trojans (b. 20, v. 38-40) [1]. Eris is the only Goddess who does not support any of the two enemies since her only preoccupation is to observe the battlefield. ("And Hate [7], the Lady of Sorrow, was gladdened to watch them. She alone of all the immortals attended this action but the other immortals were not there, but sat quietly remote and apart in their palaces, where for each one of them a house had been built in splendor along the folds of Olympos" (b. 11, v. 73-77) [1]. Secondly, Homer's Greek origin renders him a biased judge of the war. Finally, the Achaeans were trained to become the best warriors and they were famous for their martial skills due to the wars which often broke out among the different cities of Greece.

### Therapeutic interventions concerning the aforementioned injuries

**Figure 3:**
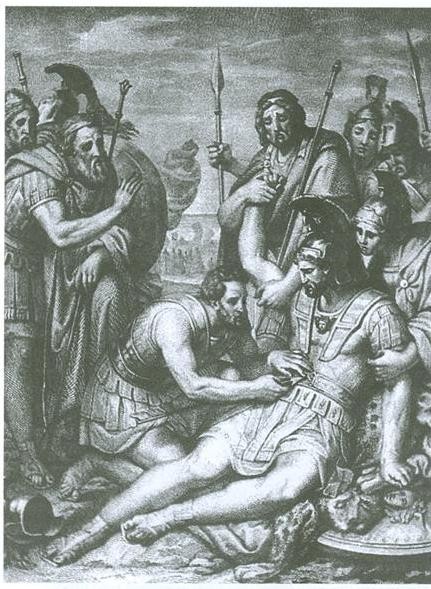
**Doctor Mahaon nurses the wound of Menelaus extracting the arrow from his chest (chalcography, F. Nenci, 1837)**.

**Figure 4:**
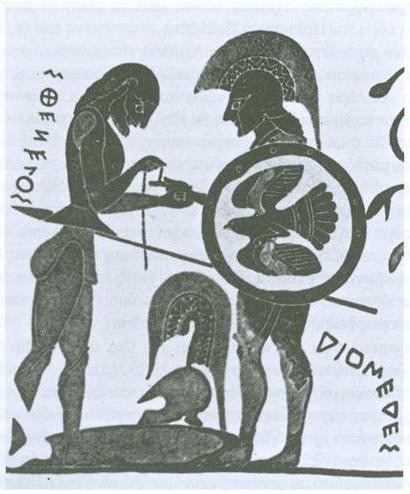
**Sthenelos nurses Diomedes' wound**. In this representation on an amphora of Chalkida (550 BC), the wound can be detected on the hand and not on the chest, as described in the original text. (b. 5, v. 111-113) [1].

It goes without saying that in a large-scale campaign like the one organized by the Achaeans the presence of doctors would have been more than necessary. Indeed, two of Asklepios' sons, Mahaon and Podaleirios, are referred to by the poet as doctors who participated in the campaign (figure 3). They also fought in the battlefield (b. 11, v. 836) [1]. In 4 cases of thoracic injuries there is a therapeutic or medical intervention. In case 2 (b. 4, v. 134-140) [1] Pandarus' arrow injures Menelaus. Venous blood gushes out of his thoracic wound ("from the cut there gushed a cloud of dark blood" (b. 4, v. 140) [1] and it is running on his thighs and calf and reached his ankles ("so, Menelaos, your shapely thighs were stained with the colour of blood, and your legs also and the ankles beneath them") (figure 4) (b. 4, v. 146-147) [1] and Agamemnon panics. He orders that they find Mahaon, son of Asklepios, who seems to have been a skilled doctor so that he could remove the arrow and use herbs which will alleviate the pain. ("But the physician will handle the wound and apply over it healing salves, by which he can put an end to the black pains") (b. 4, v. 190-191) [1]. This also demonstrates that Agamemnon was aware of the therapeutic procedure to be followed. Indeed, Mahaon first removed the arrow from the thorax and then from the flesh, which was profoundly wounded, and then he draw blood from the wound so that the venom would not enter his body. Finally, he placed therapeutic herbs over the wound, the ones which wise Cheiron had taught his father to use (b. 4, v. 213-219) [1] (figure 4). It seems that Mahaon himself was injured at some point since he was in the battlefield and fought while the battle was taking place. In case 29 (table 1) Mahaon was hit by Paris with an arrow on his right shoulder and was forced to stay outside the battlefield (b. 11, v. 505-507) [1]. The Achaeans decided to cease their charge because of their fear that Mahaon will fall into the hands of the Trojans. (b. 11, v. 508-509) [1] Idomeneus called Nestor to lead the doctor away from the battlefield to the ships (b. 11, v. 511-513) [1]. Even in the heat of the battle Idomeneus does not hesitate to praise Mahaon for his therapeutic methods since the latter can remove the arrows and use the appropriate medicines ("A healer is a man worth many men in his knowledge of cutting out arrows and putting kindly medicines on wounds") (b. 11, v. 514-515) [1]. In the same rhapsody and towards the end of it there is another reference to Mahaon's injury as well as to that of his brother, Podaleirios. Eurypylos is injured in his thigh with an arrow and he begs Patroclus to bring him into his tent so that the latter can heal him. Patroclus was taught how to heal by Achilles and since both Mahaon and Podaleirios are not available, he is the only one who can help Eurypylos. ("But help save me now at least, leading me away to my black ship, and cut the arrow out of my thigh, wash the dark blood running out of it with warm water, and put kind medicines on it, good ones, which they say you have been told of by Achilles, since Cheiron, most righteous of the Centaurs, told him about them. As for Machaon and Podaleirios, who were healers [3], I think Machaon has got a wound, and is in the shelters lying there, and himself is in need of a blameless healer, while the other in the plain is standing the bitter attack of the Trojans") (b. 11, v. 827-835) [1]. Homer describes the removal of the arrow from his thigh with Patroclus' knife, the consequent administration of medicines and the nursing of the wound (b. 11, v. 842-848) [1].

**Table 1:** The localization of the injuries of the chest according to their description (37 out of 46 injuries).

Area of the thorax	Nr of case from table 1	total
shoulder	8, 10, 11, 15, 17, 29, 35, 42, 43, 49	**10**

Interscapular area	1, 7, 9, 20, 28, 47, 50, 51, 53	**9**

breast	4, 12, 14, 19, 22, 24, 39	**7**

sternum	6, 37, 40, 41, 44, 46	**6**

clavicle	16, 48, 52	**3**

precordial area	31, 45	**2**

**Figure 5:**
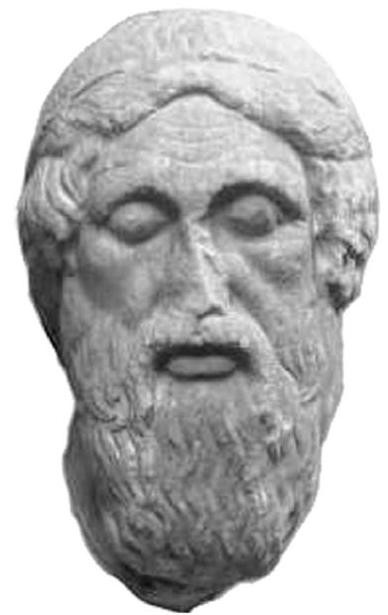
**Homer's image in marble (460 BC), Sculpture Museum of Munich**. The poet is represented as a blind man.

The third reference to an injury which received medical care was the one which was caused by Amphitryoniades (Zeus' illegitimate son) with an arrow which he threw against Hades in front of the gates of the dead (b. 5, v. 394-397) [1]. Hades resorted to Zeus and, in the end, his wound was treated by Paieon with the use of medicines from Olympos (b. 5, v. 401-2 and v. 889-890) [1]. Finally, in case 11 Pandarus' arrow is struck in Diomedes' right shoulder. The arrow penetrates his chest (we can infer that it went across the thoracic wall) and it goes out on the other side filling the chest with blood (b. 5, v. 98-100) [1]. Then Diomedes asked Sthenelos to remove the arrow (b. 5, v. 109-110) [1]. Indeed, Sthenelos dismounted from his chariot and pulled the arrow from his chest. It appears that the arrow had been firmly fixed in his chest since, when Sthenelos removed it, blood gushed from the wound and stained his tunic (b. 5, v. 111-113) [1] (figure 5).

### Discussion-Hypotheses

Homer's skillfulness and his unique talent in the narration of the events of the Trojan War are made prevalent in this epic poem. Who could present us with a more vivid picture of the mourning of Achilles' horses because of Patroclus' death? ("But the horses if Aiakides standing apart from the battle wept, as they had done since they heard how their charioteer had fallen in the dust at the hands of murderous Hektor (b. 17, v. 426-428) [1]. A blind man could not have offered such a detailed description of hunting or agricultural life in general : (b. 2, v. 467-471), (b. 11, v. 474-481), (b. 11, v. 548-555), (b. 12, v. 146-152), (b. 13, v. 471-475), (b. 13, v. 588-590), (b. 15, v. 630-636), (b. 16, v. 130-141), (b. 17, v. 657-664), (b. 18, v. 22-27) [1].

In addition, the detailed description, the detection and the symptoms of the injuries may reveal a man with knowledge of "anatomy", as well as "physiology" [2,7-10]. The detailed descriptions of the Greek doctors' interventions may demonstrate that Homer did not only have a good command of "anatomy" but he also had knowledge of "medicine" and was closely associated with the battlefield.

A plethora of medical terms, such as thumos (heart) (433 times), phrenes (chest or diaphragm) (176 times), hypochondrium (12 times), head or cranium (71 times), brain (7 times), intestines (5 times), liver (6 times) etc reinforce the idea that Homer was a knowledgeable poet [2]. There are at least 150 references to anatomical terms, mainly referring to topographic anatomy It is unlikely that a blind poet would have been able to describe the injuries using medical terms without being aware of their meaning. It may therefore be inferred that Homer was a witness of the war and that he even participated in it: he may have been one of the people appointed to nurse the wounds of the injured warriors [8-22].

## Competing interests

The authors declare that they have no competing interests.

## Authors' contributions

EA: wrote, correct and revised the manuscript. GA: translate, and revised English version. MA: did the bibliographic research found the figures. MC: did the bibliographic research and designs the manuscript. All authors read and approved the final manuscript.

## Supplementary Material

Additional file 1 **The 54 thoracic injuries are presented as they are referred to in the book and the lines in which they are found and then follow the name of the perpetrator, of the victim, the area which was injured as well as the outcome**. The severity of the injury is presented as (+), (++), or (+++) corresponding to mild, medium or severe injuries. In the last column passages from the original text are quoted and some interesting comments accompanied these parts (A = Achaean, T = Trojans, b. = book, v. = verse). Click here for file
