# Supplementary material for: The reported thoracic injuries in Homer's Iliad
Source: J Cardiothorac Surg. 2010 Nov 19;5:114. doi: 10.1186/1749-8090-5-114 (PMC2999600; doi:10.1186/1749-8090-5-114)
Supplement: Additional file 1 — The 54 thoracic injuries are presented as they are referred to in the book and the lines in which they are found and then follow the name of the perpetrator, of the victim, the area which was injured as well as the outcome. The severity of the injury is presented as (+), (++), or (+++) corresponding to mild, medium or severe injuries. In the last column passages from the original text are quoted and some interesting comments accompanied these parts (A = Achaean, T = Trojans, b. = book, v. = verse). [file 1749-8090-5-114-S1.DOC]

| **Characteristics of the case** | **Verses-Comments** |
| --- | --- |
| **Number of case : 1**  **Book** : 2  **Verses** : 265-270  **Victimizer :** Οdysseus (A)  **Victim** : Thersites (A)  **Weapon** : scepter of the  King  **Part of the body** : interscapular  area  **Causes-results** : abrasion-  pustula  **Severity :** (+) | *“so he spoke (Οdysseus) and dashed the sceptre against his back” (metathrenic) “and shoulders”-“and he doubled over, and a round tear dropped from him, and a bloody welt stood up between his shoulders under the golden scepter’s stroke, and he sat down again, frightened, in pain, and looking helplessly about wiped off the tear-drops”, “sorry though the men were they laughed over him happily, and thus they would speak to each other”* (Homer’s apparent bias against wise Agamemnon) |
| **Number of case : 2**  **Book :** 4  **Verses :** 134-140  **Victimizer :** Pandarus (Τ)  **Victim :** Menelaus (A)  **Weapon :** arrow  **Part of the body :** chest  **Causes-results :** little bleeding  – abrasion  **Severity :** (+) | *“the bitter arrow was driven against the joining of the war belt and passed clean through the war belt elaborately woven; into the elaborately wrought corselet the shaft was driven and the guard which he wore to protect his skin and keep the spears off, which guarded him best, yet the arrow plunged even through this also and with the very tip of its point it grazed the man’s skin and straightway from the cut there gushed a cloud of dark blood”*-the arrow penetrated the thoracic wall- *“first among them Zeus’ daughter, the spoiler, who standing in front of you fended aside the tearing arrow”(b. 4, v. 128-129)-“Agamemnon the lord of men was taken with shuddering fear as he saw how from the cut the dark blood trickled downward” (b. 4, v. 148-150)-“and Menelaus the warlike himself shuddered in terror” (b. 4, v. 150)^1^-“but when he saw the binding strings and the hooked barbs outside the wound”*(Homer must be referring to the cut down muscles) *“his spirit was gathered again back into him” (b. 4, v. 151-152)*- Machaon *“straightway pulled the arrow forth from the joining of the war belt, and as it was pulled out the sharp barbs were broken backwards”(b. 4, v. 213)* |
| **Number of case : 3**  **Book :** 4  **Verses :** 467-469  **Victimizer :** Aginor (Τ)  **Victim :** Elephinor (A)  **Weapon :** arrow  **Part of the body :** chest  **Causes-results :** death  **Severity :** (+++) | *“for as he hauled the corpse high-hearted Agenor, marking the ribs that showed bare under the shield as he bent over, stabbed with the bronze-pointed spear and unstrung his sinews”* |
| **Number of case : 4**  **Book :** 4  **Verses :** 473-488  **Victimizer :** Aias (A)  **Victim :** Simoeisios(Τ)  **Weapon :** spear  **Part of the body :** chest (right  breast)  **Causes-results :** death  **Severity :** (+++) | Aias *“struck him as he first came forward beside the nipple of the right breast, and the bronze spearhead drove clean through the shoulder”-“he dropped then to the ground in the dust”* |
| **Number of case : 5**  **Book :** 4  **Verses :** 527-531  **Victimizer :** Thoas (A)  **Victim :** Peiroos (Τ)  **Weapon :** javelin+sword  **Part of the body :** chest+  abdomen  **Causes-results :** death  **Severity :** (+++) | *“Thoas the aitolian hit Peiros as he ran backward with the spear in the chest above the nipple, and the bronze point fixed in the lung”-it penetrated the thoracic wall and his lung” –“and Thoas standing close dragged out the heavy spear from his chest, and drawing his sharp sword struck him in the middle of the belly, and so took the life from him”* |
| **Number of case : 6**  **Book :** 5  **Verses :** 17-24  **Victimizer :** Diomedes (A)  **Victim :** Phegeus (Τ)  **Weapon :** spear  **Part of the body :** chest  **Causes-results :** fainting  **Severity :** (+) | *“Over the left shoulder of Tydeus’ son passed the pointed spear, nor struck his body, and Diomedes thereafter threw with the bronze, and the weapon cast from his hand flew not vain but struck the chest between the nipples and hurled him from behind his horses”- “Hephaistos caught him away and rescued him, shrouded in darkness, that the aged man might not be left altogether desolate”- “but the son of high-hearted Tydeus drove off the horses and gave them to his company to lead back to the hollow vessels”* |
| **Number of case : 7**  **Book :** 5  **Verses :** 38-42  **Victimizer :**Agamemnon(A)  **Victim :** Odeon (Τ)  **Weapon :** spear  **Part of the body :** interscapular  area  **Causes-results :** death  **Severity :** (+++) | *“for in his back even as he was turning” –the javelin struck him in the interscapular area (between the shoulders) and came out of his chest on the front-“ the spear fixed between the shoulders and was driven on through the chest beyond it” - “he fell, thunderously, and his armour clattered upon him”* |
| **Number of case : 8**  **Book :** 5  **Verses :** 43-47  **Victimizer :** Idomeneus (A)  **Victim :** Phaistos (Τ)  **Weapon :** spear  **Part of the body :** right shoulder  **Causes-results :** death  **Severity :** (+++) | *“Idomeneus the spear-renowned stabbed this man just as he was mounting behind his horses, with the long spear driven in the right shoulder”-“he dropped from the chariot, and the hateful darkness took hold of him”* |
| **Number of case : 9**  **Book :** 5  **Verses :** 55-58  **Victimizer :** Menelaus (A)  **Victim :**Scamandrus(Τ)  **Weapon :** spear  **Part of the body :** interscapular  area  **Causes-results :** death  **Severity :**(+++) | *“Menelaos the spear-famed, son of Atreus, stabbed him, as he fled away before him, in the back with a spear thrust between the shoulders and driven through to the chest beyond it”*-it entered his thorax at the back and came out at the front of his body-*“he dropped forward on his face and his armour clattered upon him”-“Menelaos son of Atreus killed with the sharp spear Strophios’ son, a man of wisdomin the chase, Skamandrios, the fine huntsman of beasts. Artemis herself had taught him to strike down every wild thing that grows in the mountain forest”* |
| **Number of case : 10**  **Book :** 5  **Verses :** 79-83  **Victimizer :** Eurypylos(A)  **Victim :** Ipsinor(Τ)  **Weapon :** sword(knife)  **Part of the body :** right shoulder  **Causes-results :** death  **Severity :** (+++) | *“This man Eurypylos, the shining son of Euaimon, running in chase as he fled before him struck in the shoulder with a blow swept from the sword and cut the arm’s weight from him” –“so that the arm dropped bleeding to the ground, and the red death and destiny the powerful took hold of both eyes”* |
| **Number of case : 11**  **Book :** 5  **Verses :** 95-100  **Victimizer :** Pandaros(Τ)  **Victim :** Diomedes(A)  **Weapon :** arrow  **Part of the body :** right shoulder  **Causes-results :** injury  **Severity :** (++) | *“Now as the shining son of Lykaon, Pandaros, watched him storming up the plain scattering the battalions before him, at once he strained the bent bow against the son of Tydeus, and shot”- “and hit him as he charged forward, in the right shoulder at the hollow of the corselet”* : the arrow struck his right shoulder and it penetrated the cavity of his chest (it must have moved across the wall)- *“and the bitter arrow went straight through holding clean to its way, and the corselet was all blood-spattered” –“Sthenelos sprang to the ground from his chariot and standing beside him pulled the sharp arrow clean through his shoulder and the blood shot up spurting through the delicate tunic” (b. 5, v. 111-113)* |
| **Number of case : 12**  **Book :** 5  **Verses :** 144-145  **Victimizer :** Diomedes(A)  **Victim :** Astynoos (Τ)  **Weapon :** spear  **Part of the body :** chest (breast)  **Causes-results :** death  **Severity :** (+++) | *“he killed Astynoos… striking one with the bronze-heeled spear above the nipple”* |
| **Number of case : 13**  **Book :** 5  **Verses :** 280-282  **Victimizer :** Pandarus (Τ)  **Victim :** Diomedes (A)  **Weapon :** spear  **Part of the body :** chest  **Causes-results :** injury  **Severity :** (++) | *“So he spoke, and balanced the spear far-shadowed, and threw it, and struck the son of Tydeus in the shield, and the flying bronze spearhead was driven clean through and into the corselet”*-the spear was planted in his thoracic wall |
| **Number of case : 14**  **Book :** 5  **Verses :** 392-395  **Victimizer :** Amphitryoni-  ades (Zeus’  son ?)  **Victim :** Hera(Goddess)  **Weapon :** arrow  **Part of the body :** right chest  **Causes-results :** injury  **Severity :** (++) | Healed injury –*Hera had to endure it when the strong son of Amphitryon struck her beside the right breast with a tri-barbed arrow, so that the pain he gave her could not be quieted* |
| **Number of case : 15**  **Book :** 5  **Verses :** 395-400  **Victimizer :** Amphitryoni-  ades (Zeus’  son?)  **Victim :** Hades (God)  **Weapon :** arrow  **Part of the body :** shoulder  **Causes-results :** injury  **Severity :** (++) | Healed injury-Hades the gigantic had to endure with the rest the flying arrow when this self-same man, the son of Zeus of the aegis, struck him among the dead men at Pylos, and gave him to agony –*“but Paieon, scattering medicines that still pain, healed him, since he was not made to be one of the mortals” (b. 5, v. 401-402)* |
| **Number of case : 16**  **Book :** 5  **Verses :** 578-579  **Victimizer :** Menelaus (A)  **Victim :** Pylaimenes(Τ)  **Weapon :** spear  **Part of the body :** clavicle  **Causes-results :** death  **Severity :** (+++) | *“Menelaus the spear-famed, son of Atreus, stabbed him with the spear as he stood his ground, and struck the collar-bone”*: Menelaus struck Pylaimenes in the clavicle, thus it may be inferred that he died of massive haemorrhage of clavicular vessels or of severe injury of the lung |
| **Number of case : 17**  **Book :** 7  **Verses :** 13-16  **Victimizer :** Glaukos (Τ)  **Victim :** Iphinoos (A)  **Weapon :** spear  **Part of the body :** shoulder  **Causes-results :** death  **Severity :** (+++) | *“And Glaukos, lord of the Lykian men, the son of Hippolochos, struck down with the spear Iphinoos in the strong encounter, Dexias’ son, as he leapt up behind his fast horses, striking him in the shoulder. He dropped from car to ground, and his limbs’ strength was broken”* |
| **Number of case : 18**  **Book :** 7  **Verses :** 268-272  **Victimizer :** Αeas (A)  **Victim :** Hector (Τ)  **Weapon :** stone  **Part of the body :** chest +  abdomen  **Causes-results :** concussion,  dizziness  S**everity :** (+) | *“Aias in turn lifting a stone far greater whirled it and threw, leaning into the cast his strength beyond measure, and the shield broke inward under the stroke of the rock like a millstone, and Hector’s very knees gave, so that he sprawled backward, shield beaten upon him” Apollo intervened and “lifted him upright” (b. 7, v. 272)* |
| **Number of case : 19**  **Book :** 8  **Verses :** 118-122  **Victimizer :** Diomedes (A)  **Victim :** Eniopeus (Τ)  **Weapon :** spear  **Part of the body :** chest (breast)  **Causes-results :** death  **Severity :** (+++) | Diomedes, *“as he raged straight forward the son of Tydeus threw at him and missed his man, but struck the charioteer, his henchman, Eniopeus, the son of high-hearted Thebaios , striking him in the chest next to the nipple as he gripped the reins of his horses. He fell out of the chariot, and the fast-footed horses shied away. And there his life and his strength were scattered.”* |
| **Number of case : 20**  **Book :** 8  **Verses :** 257-260  **Victimizer :** Diomedes (A)  **Victim :** Agelaos (Τ)  **Weapon :** spear  **Part of the body :** interscapular  area  **Causes-results :** death  **Severity :** (+++) | *“For in his back even as he was turning the spear fixed between the shoulders and was driven on through the chest beyond it”: the spear was planted in the interscapular area-“he fell from the chariot, and his armour clattered upon him”* |
| **Number of case : 21**  **Book :** 8  **Verses :** 300-308  **Victimizer :** Teucros (A)  **Victim :** Gorgythion(Τ)  **Weapon :** arrow  **Part of the body :** chest  **Causes-results :** death  **Severity :** (+++) | *“He spoke, and let fly another shaft from the bowstring, straight for Hector, and all his heart was straining to hit him; but missed his man, and struck down instead a strong son of Priam, Gorgythion the blameless, hit in the chest by an arrow; Gorgythion whose mother was lovely Kastianeira, Priam’s bride from Aisyme, with the form of a goddess. He bent drooping his head to one side, as a garden poppy bends beneath the weight of its yield and the rains of springtime; so his head bent slack to one side beneath the helm’s weight”* |
| **Number of case : 22**  **Book :** 8  **Verses :** 309-315  **Victimizer :** Teukros (A)  **Victim :** Αrcheptole-  mos (Τ)  **Weapon :** arrow  **Part of the body :** chest  **Causes-results :** death  **Severity :** (+++) | *“But Teukros now let fly another shaft from the bowstring, straight for Hector, and all his heart was straining to hit him, yet missed his man once again as Apollo faltered his arrow, and struck Archeptolemos, bold charioteer of Hector, in the chest next to the nipple as he charged into the fighting. He fell out of the chariot, and the fast-footed horses shied away. And there his life and his strength were scattered. And bitter sorrow closed over Hector’s heart for his driver, yet grieving as he did for his friend he left him to lie there, and called to his brother Kebriones who stood near to take up the reins of the horses, nor did he disobey him”* |
| **Number of case : 23**  **Book :** 8  **Verses :** 320-329  **Victimizer :** Hector (Τ)  **Victim :** Teukros (A)  **Weapon :** stone  **Part of the body :** clavicle-  thorax-neck  **Causes-results :** injury  **Severity :** (++) | *“But Hector himself vaulted down to the ground from the shining chariot crying a terrible cry and in his hand caught up a great stone, and went straight for Teukros, heart urgent to hit him. Now Teukros had drawn a bitter arrow out of his quiver, and laid it along the bowstring, but as he drew the shaft by his shoulder, there where between neck and chest the collar-bone interposes, and this is a spot most mortal; in this place shining-helmed Hector struck him in all his fury with the jagged boulder, smashing the sinew and all his arm at the wrist was deadened”*: the stone broke his brachial nexus |
| **Number of case : 24**  **Book :** 11  **Verses :** 107-108  **Victimizer :**Agamemnon(A)  **Victim :** Isos (Τ)  **Weapon :** spear  **Part of the body :** chest  **Causes-results :** death  **Severity :** (+++) | *“The bastard, Isos, was charioteer and renowned Antiphos rode beside him…This time the son of Atreus, wide-powerful Agamemnon struck Isos with the strown spear in the chest above the nipple and hit Antiphos by the car with the sword and hurled him from his horses”* |
| **Number of case : 25**  **Book :** 11  **Verses :** 143-144  **Victimizer :**Agamemnon(A)  **Victim :** Peisandros (Τ)  **Weapon :** spear  **Part of the body :** chest  **Causes-results :** death  **Severity :** (+++) | *“Next he caught Peisandros and Hippolochos stubborn in battle, sons of Antimachos the wise, who beyond all others had taken the gold of Alexandros, glorious gifts, so that he had opposed the return of Helen to fair-haired Menelaos. Poweful Agamemnon caught his two sons riding in one chariot, who together guided the running horses. Now the glittering reins escaped from the hands of both of them and they were stunned with fear, for against them rose like a lion Atreus’ son, and they supplicated him out of the chariot” (b. 11, v. 122-129) but Agamemnon “spurned Peisandros to the ground from the chariot with a spear-stroke in the chest, and he raised on his back to the ground.”(b. 11, v. 143-144)* |
| **Number of case : 26**  **Book :** 11  **Verses :** 259-261  **Victimizer :** Agamemnon(A)  **Victim :** Koon (Τ)  **Weapon :** spear and then a  sword  **Part of the body :** chest and head  **Causes-results :** decapitation  **Severity :** (+++) | *“He (Koon) came from the side and unobserved at great Agamemnon and stabbed with his spear at the middle arm, underneath the elbow, and the head of the glittering spear cut its way clean through. Agamemnon the lord of men shuddered with fear then but even so did not give up the attack or his fighting but sprang at Koon, gripping a spear that struck with the wind’s speed. Now Koon was dragging his father’s son, his brother Iphidamas, by the foot back eagerly, and cried out on all the bravest, but as he dragged him into the crowd, Agamemnon thrust at him with the smoothed bronze spear underneath the knobbed shield, and unstrung him, then came up and hewed off his head over Iphidamas.” (b. 11, v. 251-261)* |
| **Number of case : 27**  **Book :** 11  **Verses :** 434-438  **Victimizer :** Sokos (Τ)  **Victim :** Odysseus (A)  **Weapon :** spear  **Part of the body :** chest  **Causes-results :** injury  **Severity :** (++) | *“He spoke, and stabbed Odysseus’ shield in its perfect circle. All the way through the glittering shield went the heavy spearhead and crashed its way through the intricately wrought corselet, and all the skin was torn away from his ribs, yet Pallas Athene would not let the point penetrate the man’s vitals”*: the spear reached the intercostal area, without however entering the body |
| **Number of case : 28**  **Book :** 11  **Verses :** 446-449  **Victimizer :** Odysseus (A)  **Victim :** Sokos (Τ)  **Weapon :** spear  **Part of the body :** interscapular  **Causes-results :** death  **Severity :** (+++) | “He spoke, and Sokos turning from him was striding in flight but in his back even as he was turning the spear fixed between the shoulders and was driven on through the chest beyond it. He fell, thunderously, and great Odysseus boasted over him”, then Odysseus *“dragged the heavy spear of wise Sokos out of his flesh and out of the shield massive in the middle, and as it was torn out the blood sprang and his heart was sickened”* |
| **Number of case : 29**  **Book :** 11  **Verses :** 505-507  **Victimizer :** Paris (Τ)  **Victim :** Mahaon (A)  **Weapon :** arrow  **Part of the body :** shoulder  **Causes-results :** injury  **Severity :** (++) | *“Yet even so the Achaeans would not have given from his path had not Alexandros, the lord of lovely-haired Helen, stayed from his beavery the shepherd of the people, Machaon, hitting him with a three-barbed arrow in the right shoulder”*- although he was not killed, he withdrew from battle because *“the Achaeans whose wind was fury were frightened for him, that the enemy might catch him in the back turn of the fighting”* |
| **Number of case : 30**  **Book :** 13  **Verses :** 183-187  **Victimizer :** Hector (T)  **Victim :** Amphimachos(A)  **Weapon :** spear  **Part of the body :** chest  **Causes-results :** sudden death  **Severity :** (+++) | *“After killing Imbrios, Teukros attempted to despoil him and Hector threw his javelin at him. Instead, he struck Amphimachos in the chest - He fell on the ground with a thud and his armour fell on him”* |
| **Number of case : 31**  **Book :** 13  **Verses :** 434-440  **Victimizer :** Idomeneus(A)  **Victim :** Alkathoos (Τ)  **Weapon :** spear  **Part of the body :** chest (sternum)  **Causes-results :** death  **Severity :** (+++) | *“But now Poseidon beat him down at the hands of Idomeneus, for he bewitched his shining eyes, made moveless his bright limbs, so that he could not run backward, neither evade him, but stood like a statue or a tree with leaves towering motionless, while fighting Idomeneus stabbed at the middle of his chest with the spear, and broke the bronze armour about him which in time before had guarded his body from destruction.”* |
| **Number of case : 32**  **Book :** 13  **Verses :** 506-508  **Victimizer :** Idomeneus(A)  **Victim :** Oinomaos (Τ)  **Weapon :** spear  **Part of the body :** chest+  abdomen  **Causes-results :** death  **Severity :** (+++) | *“Idomeneus hit Oinomaos in the middle belly (abdomen) and broke the hollow of the corselet, so that the entrails spurted from the bronze, and he fell clawing the dust in his fingers.”* |
| **Number of case : 33**  **Book :** 13  **Verses :** 516-520  **Victimizer :** Deiphobos (Τ)  **Victim :** Askalaphos (A)  **Weapon :** spear  **Part of the body :** shoulder+  chest  **Causes-results :** death  **Severity :** (+++) | *“As he backed slowly Deiphobos made a cast with the shining spear, since he held a fixed hatred forever against him (Idomeneus), but missed him yet once again and struck down with the spear the war god’s son Askalaphos, so that the powerful spear was driven through his shoulder, and he dropping in the dust clawed the ground in his fingers”*-Askalaphos was probably injured in his chest since his death was instantaneous |
| **Number of case : 34**  **Book :** 14  **Verses :** 410-420  **Victimizer :** Aeas (A)  **Victim :** Hector (Τ)  **Weapon :** stone  **Part of the body :** chest + neck  **Causes-results :** dizziness-  concussion  **Severity :** (+) | *“But as he drew away huge Telamonian Aeas caught up a rock; there were many, holding- stones for the fast ships, rolled among the feet of the fighters; he caught up one of these and hit him in the chest next to the throat over his shield rim, and spun him around like a top with the stroke, so that he staggered in a circle” (b. 14, v. 409-414)-so Hector in all his strength dropped suddenly in the dust, let fall the spear from his hand, and his shield was beaten upon him, and the helm, and his armor elaborate with bronze clashed over him” (b. 14, v. 418-420)* |
| **Number of case : 35**  **Book :** 14  **Verses :** 449-452  **Victimizer :** Polydamas (Τ)  **Victim :** Prothoenor (A)  **Weapon :** spear  **Part of the body :** right shoulder  **Causes-results :** death  **Severity :** (+++) | *“Polydamas of the shaken spear came up to stand by him, Panthoos’ son, and struck in the right shoulder(apparently the spear penetrated his thorax) Prothoenor son of Areilykos, and the powerful spear was driven through the shoulder, and he dropping in the dust clawed the ground in his fingers” (b. 14, v. 449-452)*-Polydamas vaunted terribly over him, calling in a great voice” (b. 14, v. 453) and *“he stirred the anger in wise Telamonian Aeas”(b. 14, v. 459)* |
| **Number of case : 36**  **Book :** 15  **Verses :** 419-423  **Victimizer :** Aeas(A)  **Victim :** Kaletor (Τ)  **Weapon :** spear  **Part of the body :** chest  **Causes-results :** death  **Severity :** (+++) | *“Shining Aeas struck with spear Kaletor, Klytios’ son, in the chest(thorax) as he brought fire to the vessel. He fell, thunderously, and the torch dropped from his hand”* |
| **Number of case : 37**  **Book :** 15  **Verses :** 520-524  **Victimizer :** Meges (A)  **Victim :** Kroismos (Τ)  **Weapon :** spear  **Part of the body :** chest(sternum)  **Causes-results :** death  **Severity :** (+++) | Meges attempted to kill Polydamas but Apollo wouldn’t permit it : *“Meges seeing it lunged at him, but Polydamas bent down and away, so that Meges missed him. Apollo would not let Panthoos’ son go down among the front fighters, but Meges stabbed with the spear the middle of the chest of Kroismos.”* |
| **Number of case : 38**  **Book :** 15  **Verses :** 541-543  **Victimizer :** Menelaus (A)  **Victim :** Dolops (Τ)  **Weapon :** spear  **Part of the body :** shoulder-  chest  **Causes-results :** death  **Severity :** (+++) | *“Yet Dolops stood his ground and fought on, in hope still of winning, but meanwhile warlike Menelaos came to stand beside Meges, and came from the side and unobserved with his spear, and from behind threw at his shoulder, so the spear tore through his chest in its fury to drive on, so that Dolops reeled and went down, face forward”*: the spear penetrated his thorax and went out from his sternum |
| **Number of case : 39**  **Book :** 15  **Verses :** 575-584  **Victimizer :** Antilochos(A)  **Victim :** Melanippos(Τ)  **Weapon :** arrow  **Part of the body :** breast  **Causes-results :** death  **Severity :** (+++) | *“He made no vain cast but struck Hiketaon’s son, Melanippos, the high-hearted, in the chest next to the nipple as he swept into the fighting. He fell, thunderously, and darkness closed over both eyes. Antilochos sprang forth against him, as a hound rushes against a stricken fawn that as he broke from his covert a hunter has shot at, and hit, and broken his limbs’ strength. So Antilochos stubborn in battle sprang, Melanippos, at you, to strip your armor, but did not escape brilliant Hektor’s notice, who came on the run through the fighting against him.”*-Homer changes from third to second person in his description, which is a sign of respect on the part of the author |
| **Number of case : 40**  **Book :** 15  **Verses :** 645-652  **Victimizer :** Hector (Τ)  **Victim :** Periphetes (A)  **Weapon :** spear  **Part of the body :** chest(sternum)  **Causes-results :** death  **Severity :** (+++) | *“For as he whirled about to get back, he fell over the out-rim of the shield he carried, which reached to his feet to keep the spears from him. Stumbling on this he went over on his back, and the helmet that circled his temples clashed horribly as he went down. Hector saw it sharply, and ran up and stood beside him, and stuck the spear into his chest and killed him before the eyes of his dear friends, who for all their sorrowing could do nothing to help their companion, being themselves afraid of great Hector”* |
| **Number of case : 41**  **Book :** 16  **Verses :** 311-312  **Victimizer :** Menelaus (A)  **Victim :** Thoas (Τ)  **Weapon :** lance and/or  spear?  **Part of the body :** sternum  **Causes-results :** death  **Severity :** (+++) | *“Meanwhile warlike Menelaos stabbed Thoas in the chest where it was left bare by the shield, and unstrung his limbs’ strength”* |
| **Number of case : 42**  **Book :** 16  **Verses :** 319-325  **Victimizer :** Thrasymedes(A)  **Victim :** Maris (Τ)  **Weapon :** spear  **Part of the body :** head of the  humerus-  shoulder  **Causes-results :** death  **Severity :** (+++) | *“Maris with the spear from close up made a lunge at Antilochos in rage for his brother standing in front of the corpse*” (probably because he wanted to ensure that the victim would not be stripped off his armour or because he intended to strike Antilochos) , *“but before him godlike Thrasymedes was in with a thrust before he could stab, nor missed his quick stroke into the shoulder, and the spearhead shore off the arm’s base clear away from the muscles and torn from the bone utterly”*: the spear struck him in the biceps and he cut off the muscles from the bone, apparently segmenting the brachial artery (I regard hemorrhage as the most probable cause of his death-both brothers died together fighting and they were both killed by two brothers, the sons of King Nestor!) |
| **Number of case : 43**  **Book :** 16  **Verses :** 342-344  **Victimizer :** Meriones (A)  **Victim :** Akamas (Τ)  **Weapon :** spear  **Part of the body :** shoulder  **Causes-results :** death  **Severity :** (+++) | While Akamas was attempting to get on the chariot and escape, Meriones struck him (Homer does not specify the weapon) in the shoulder: *“Meriones on his light feet overtaking Akamas stabbed him in the right shoulder as he climbed up behind his horses and the darkness drifted over his eyes as he crashed from the chariot”* |
| **Number of case : 44**  **Book :** 16  **Verses :** 394-401  **Victimizer :** Patroclos (A)  **Victim :** Pronoos (Τ)  **Weapon :** spear  **Part of the body :** sternum  **Causes-results :** death  **Severity :** (+++) | *“But Patroklos, when he had cut away their first battalions, turned back to pin them against the ships, and would not allow them to climb back into their city though they strained for it, but sweeping through the space between the ships, the high wall, and the river, made havoc and exacted from them the blood price for many”* |
| **Number of case : 45**  **Book :** 16  **Verses :** 477-483  **Victimizer :** Patroclos (A)  **Victim :** Sarpedon (Τ)  **Weapon :** spear  **Part of the body :** chest  (precordia area)  **Causes-results :** death  **Severity :** (+++) | *“Once again Sarpedon threw wide with a cast of his shining spear, so that the pointed head overshot the left shoulder of Patroklos; and now Patroclos made the second cast with the brazen spear, and the shaft escaping his hand was not flung vainly but struck where the beating heart is closed in the arch of the muscles”*: Patroclos struck him where the diaphragm meets the heart- *“he fell, as when an oak goes down or a white poplar, or like a towering pine tree which in the mountains the carpenters have hewn down with their whetted axes to make a ship-timber. So he lay there felled in front of his horses and chariots roaring, and clawed with his hands at the bloody dust” (b. 16, v. 477-486)*- *“he spoke, and as he spoke death’s end closed over his nostrils and eyes, and Patroclos stepping heel braced to chest dragged the spear out of his body, and the midriff came away with it so that he drew out with the spearhead the life of Sarpedon, and the Myrmidons close by held in the hard-breathing horses as they tried to bolt away, once free of their master’s chariot” b. 16, v. 502-507)* |
| **Number of case : 46**  **Book :** 16  **Verses :** 593-601  **Victimizer :** Glaucos (Τ)  **Victim :** Bathycles (A)  **Weapon :** spear  **Part of the body :** chest  (sternum)  **Causes-results :** death  **Severity :** (+++) | *“But Glaucos was first, lord of the shield-armoured Lykians, to turn again, and killed Bathykles the great-hearted, beloved son of Chalkon, who had dwelled in his home in Hellas conspicuous for wealth and success among all the Myrmidons. It was he whom Glaucos stabbed in the middle of the chest, turning suddenly back with his spear as he overtook him. (apparently, his sternum was left unprotected)He fell, thunderously, and the closing sorrow came over the Achaeans as the great man went down, but the Trojans were gladdened greatly and came and stood in a pack about him”* |
| **Number of case : 47**  **Book :** 16  **Verses :** 791-809  **Victimizer :** Euphorbos (Τ)  **Victim :** Patroclos (A)  **Weapon :** javelin  **Part of the body :** interscapular  area  **Causes-results :** injury  **Severity :** (++) | *“there, Patroclos, the end of your life was shown forth, since Phoibos came against you there in the strong encounter dangerously”*(b. 16, v. 787-789)- *“Phoibos Apollo now struck away from his head the helmet four-horned and hollow-eyed, and under the feet of the horses it rolled clattering, and the plumes above it were defiled by blood and dust.”*(b. 16, v. 792-797)- *“the lord Apollo, son of Zeus, broke the corselet upon him. Disaster caught his wits, and his shining body went nerveless. He stood stupidly, and from close behind his back a Dardanian man hit him between the shoulders (interscapular area) with a sharp javelin: Euphorbos, son of Panthoos, who surpassed all men of his own age with the throwing spear, and in horsemanship and the speed of his feet.”*(b. 16, v. 804-809)- *“he first hit you with a thrown spear, o rider Patroclos, nor broke you, but ran away again, snatching out the ash spear from your body, and lost himself in the crowd, not enduring to face Patroclos, naked as he was, in close combat. Now Patroclos, broken by the spear and the god’s blow, tried to shun death and shrink back into the swarm of his own companions”*(b. 16, v. 812-817) |
| **Number of case : 48**  **Book :** 17  **Verses :** 304-311  **Victimizer :** Hector (Τ)  **Victim :** Schedios (A)  **Weapon :** javelin  **Part of the body :** clavicle  **Causes-results :** death  **Severity :** (+++) | *“Again Hector threw at Aeas with the shining javelin, but Aeas with his eyes straight on him avoided the bronze spear by a little, and Hector struck Schedios, the son of high-hearted Iphitos and far the best of phokians, one who lived in his home in famous Panopeus and was lord over many people. He struck him fair beneath the collar-bone(in the thorax), and the pointed bronze head tore clean through and came out by the base of the shoulder”* |
| **Number of case : 49**  **Book :** 17  **Verses :** 593-600  **Victimizer :** Polydamas (Τ)  **Victim :** Peneleos (A)  **Weapon :** spear  **Part of the body :** shoulder  **Causes-results :** injury  **Severity :** (++) | *“And now the son of Kronos caught up the betasselled glaring aegis, and shrouded Ida in mists. He let go a lightning flash and a loud thunder stroke, shaking the mountain, gave victory to the Trojans, and terrified the Achaeans. First to begin the flight was Peneleos the Boeotian. For he, turning always toward the attack, was hit in the shoulder’s end, a slight wound, but the spear of Polydamas, who had thrown it from a stance very close to him, had grated the bone’s edge”* (the brachial or clavicle) |
| **Number of case : 50**  **Book :** 20  **Verses :** 401-406  **Victimizer :** Achilles (A)  **Victim :** Hippodamas (Τ)  **Weapon :** spear  **Part of the body :** back  (interscapular area)  **Causes-results :** death  **Severity :** (+++) | *“Next he stabbed with a spear-stroke in the back Hippodamas as he fled away before him and sprang from behind his horses. He blew his life away, bellowing, as when a bull bellows as he is dragged for Poseidon, lord of Helike, and the young men drag him. In such bulls the earth shaker glories. Such was his bellowing as the proud spirit flitted from his bones.”* |
| **Number of case : 51**  **Book :** 20  **Verses :** 487-489  **Victimizer :** Achilles (A)  **Victim :** Areithoos (Τ)  **Weapon :** spear  **Part of the body :** back  (interscapular  area)  **Causes-results :** death  **Severity :** (+++) | *“He dropped from the chariot, but as Areithoos his henchman turned the horses away Achilles stabbed him with the sharp spear in the back, and thrust him from the chariot. And the horses bolted.”* |
| **Number of case : 52**  **Book :** 21  **Verses :** 116-120  **Victimizer :** Achilles (A)  **Victim :** Mykaon (Τ)  **Weapon :** sword  **Part of the body :** clavicle  **Causes-results :** death  **Severity :** (+++) | *“He let go of the spear and sat back, spreading wide both hands; but Achilles drawing his sharp sword struck him beside the neck at the collar-bone, and the double-edged sword plunged full length inside. He dropped to the ground, face downward, and lay at length, and the black blood flowed, and the ground was soaked with it. Achilles caught him by the foot and slung him into the river”* |
| **Number of case : 53**  **Book :** 21  **Verses :** 403-408  **Victimizer :** Athene  (Goddess)  **Victim :** Ares (God)  **Weapon :** Stone  **Part of the body :** interscapular  area-neck  **Causes-results :** injury- falling  down  **Severity :** (+) | *“There blood-dripping Ares made his stab with the long spear, but Athena giving back caught up in her heavy hand a stone that lay in the plain, black and rugged and huge, one which men of a former time had set there as boundary mark of the cornfield. With this she hit furious Ares in the neck, and unstrung him. He spread over seven acres in his fall, and his hair dragged in the dust, and his armour clashed.”* |
| **Number of case : 54**  **Book :**21  **Verses :** 423-425  **Victimizer :** Athene  (Goddess)  **Victim :** Aphrodite  (Goddess)  **Weapon :** hand  **Part of the body :** breast  **Causes-results :** injury-falling  down  **Severity :** (+) | *“She spoke, and Athene swept in pursuit, heart full of gladness, and caught up with her and drove a blow at her breasts with her ponderous hand, so that her knees went slack and the heart inside her.”* |

***Additional file: the 54 thoracic injuries are presented as they are referred to in the book and the lines in which they are found and then follow the name of the perpetrator, of the victim, the area which was injured as well as the outcome. The severity of the injury is presented as (+), (++), or (+++) corresponding to mild, medium or severe injuries. In the last column passages from the original text are quoted and some interesting comments accompanied these parts ( A= Achaean, T=Trojans, b.=book, v.=verse).***
